# Occurrence of *Myxobolus* spp. (Myxozoa) in the blood of *Metynnis lippincottianus* (Osteichthyes: Serrasalmidae) from eastern Amazon, Brazil

**DOI:** 10.1590/S1984-29612024022

**Published:** 2024-05-17

**Authors:** Nyelle Priscila Brito Façanha, Rafaela Franco de Araújo, Aldi Feiden, Géssica Laila Matos da Silva, Maria Danielle Figueiredo Guimarães Hoshino, Eliane Tie Oba Yoshioka, Marcela Nunes Videira

**Affiliations:** 1 Universidade Federal do Amapá – UNIFAP, Macapá, AM, Brasil; 2 Universidade Estadual do Oeste do Paraná – UNIOESTE, Toledo, PR, Brasil; 3 Universidade do Estado do Amapá – UEAP, Macapá, AP, Brasil; 4 Empresa Brasileira de Pesquisa Agropecuária – EMBRAPA, Macapá, AP, Brasil

**Keywords:** Fish, Serrasalmidae, hemoparasite, Myxosporea, Peixe, Serrasalmidae, hemoparasito, Myxosporea

## Abstract

Myxozoans are obligatory parasites and can be found in various organs and bloodstreams of fish, thus, the objective of this work was to describe the occurrence of *Myxobolus* spp. in the circulating blood of *Metynnis lippincottianus* from River Curiaú, Macapá City, eastern Amazon, Brazil. The samples of *M. lippincottianus* (11) were caught using cast net and gillnets. The fish blood was collected by puncturing the caudal vessel, using needles and syringes containing 10% of EDTA solution. Blood smear were prepared and panchromatic stained with a combination of May Grunwald-Giemsa-Wright, for observation and examination of the parasitic structures in optical microscope. Tissues from the kidney was examined using specific stereoscopic binoculars to check for the presence of cysts, lesions and parasites. The prevalence of *Myxobolus* spp. infecting the circulating blood of the fish was 36.36% (4/11) and 15 spores of mixosporyds were visualized. *Myxobolus* spp. had a prevalence of 54.55% (6/11) in host's kidney tissue and the morphometric spores data converge with observed in the blood. The morphological characteristics of the spores in the blood samples revealed two morphotypes of *Myxobolus* spp. This is the sixth occurrence recorded of *Myxobolus* spp. infecting fish blood in Brazil.

## Resumo

Mixozoários são parasitos obrigatórios e podem ser encontrados em diversos órgãos e corrente sanguínea dos peixes. Objetivou-se neste trabalho descrever a ocorrência de *Myxobolus* spp. no sangue circulante de *Metynnis lippincottianus,* oriundo do rio Curiaú, cidade de Macapá, Amazônia Oriental, Brasil. Os exemplares de *M. lippincottianus* (n=11) foram capturados, usando-se tarrafas e redes de emalhar. O sangue de cada peixe foi coletado por punção do vaso caudal, com o auxílio de agulhas e seringas contendo EDTA 10%. Após, realizaram-se extensões sanguíneas e coloração pancromática com May Grünwald-Giemsa-Wright, para a observação e exame das estruturas parasitárias em microscópio óptico. Tecidos do rim foram examinados, utilizando-se estereomicroscópio binocular para checar a presença de cistos, lesões e parasitos. A prevalência de *Myxobolus* spp., infectando o sangue circulante dos peixes, foi de 36,36% (4/11), e um total de 15 esporos de mixosporídeos foram visualizados. *Myxobolus* spp. teve prevalência de 54,55% (6/11) no tecido renal do hospedeiro, e os dados morfométricos dos esporos convergem com os observados no sangue. As características morfológicas dos esporos, nas amostras de sangue evidenciaram dois morfotipos de *Myxobolus* spp. Esse é o sexto registro de ocorrência de *Myxobolus* spp. infectando o sangue de peixes no Brasil.

## Introduction

Parasitism in fish is a common phenomenon in natural and farmed aquatic environments, where various species of parasites can infect and affect the health of their hosts. These parasites can be found in various parts of the fish body, such as the gills, muscles, skin, intestines, liver, and blood, where they can cause various diseases. Some parasites can infect a wide range of hosts whereas others are specific to both the species of fish and the body tissues infected (Molnár, 2002; Sitjà-Bobadilla, 2008; Ferreira et al., 2021).

*Metynnis lippincottianus* Cope, 1870, is a freshwater pelagic fish, with a wide distribution in South American water systems, including the drainages of French Guiana, the basins of the lower and middle Amazon and the drainages of northeastern Brazil (Zarske & Géry, 1999; Froese & Pauly, 2023). Popularly known as “pratinha”, “pacu,” and “pacu-redondo”, *M. lippincottianus* has a generalist feeding behavior, with a diet based on filamentous algae, terrestrial and aquatic plants, detritus, microcrustaceans and ostracod molluscs (Hoshino & Tavares-Dias, 2014; Kliemann et al., 2022), and it belongs to the Serrasalmidae family, which is important in the Brazilian aquarium trade as ornamental fish, as well as part of the diet of riverside populations in the Amazon region (Moreira et al., 2009; Yamada et al., 2012).

Fish like *M. lippincottianus* can host distinct species of parasites, such as the *Myxobolus* genus, which has the greatest number of species. Some species of this genus are known to be pathogenic, causing specific diseases in fry of regional and exotic fish species, including “whirling disease” in salmonids and myxobolosis in certain round fish (Steinbach Elwell et al., 2009). The clinical signs can vary according to the fish species host and the location of infection (Békési et al., 2002; Oliveira et al., 2021; Capodifoglio et al., 2019).

This type of infection can cause various problems for the host fish, and the clinical signs can compromise their health and well-being. Another associated problem is parasite transmission to other fish species or even adaptation to new infection sites. This can cause pathologies, lack of infection control, and spread of infection, increasing the risk of parasitic disease outbreaks in fish populations, causing greater problems and even an imbalance in host-linked biodiversity (Santos et al., 2013; Farias et al., 2021). Thus, fish parasitology is an important area of study for understanding the epidemiology of parasitic infections in fish, as well as for developing strategies to prevent and control parasitic diseases in aquatic environments.

In Brazil, studies about the occurrence of myxozoans infecting the blood of fish have been carried out in several regions (Central-West and North), with spores (myxospore phase) of the genus *Myxobolus* being the most common (Paperna & Di Cave, 2001; Maciel et al., 2011; Úngari et al., 2021). Also, *Sphaerospora* sp. were identified as parasitizing Amazonian fish. In Europe and Asia, other parasites of the genus *Chloromyxum*, *Myxobolus* and *Sphaerospora* have been detected in the kidney and blood of fish (Yang et al., 2022; Holzer et al., 2006; Baska & Molnar, 1988; Lom et al., 1985).

In order to contribute to information on hemoparasitism linked *Myxobolus* species in Amazonian fish, this study aimed to describe the occurrence of two morphotypes of this parasite in the circulating blood of *M. lippincottianus* (Osteichthyes: Serrasalmidae) from the Curiaú River, Macapá, Amapá, Eastern Amazon, Brazil.

## Material and Methods

### Collection and transportation of biological material

**Figure 1:**
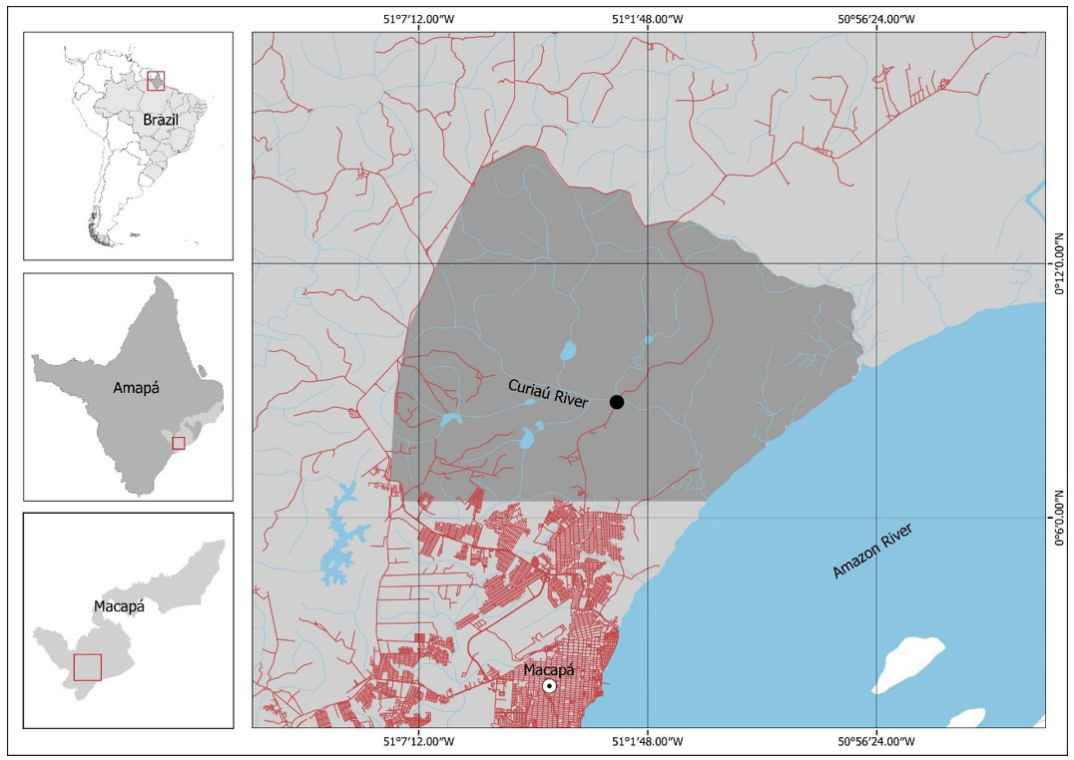
Brazil map with Amapá state, highlighting the Curiaú River Environmental Protection Area and its hydrography (gray line). The dot indicates the location where *Metynnis lippincottianus* specimens were captured.

*Metynnis lippincottianus (*n = 11) specimens were collected from the Curiaú River Environmental Protection Area (APA do Rio Curiaú) (0°8'43.6” N, 51°2'30.3” W) (Figure 1). The Curiaú River APA covers the Curiaú River basin, which has an area of approximately 584.47 km^2^, a drainage system interconnected by intermittent and perennial lakes, and the influence of the tidal and rainfall regime (Brito et al., 2022).

The fish were caught using cast net and gillnets with varying mesh sizes, to obtain a significant sample of individuals with different sizes and body weights. The fish were placed in plastic bags containing water from their own habitat, aerated using battery-operated aerators. The animals were then transported alive to the Laboratory of Morphophysiology and Animal Health - LABMORSA / UEAP, Macapá, AP.

### Parasitological analysis

In the laboratory, the animals were kept in aquariums with constant aeration until further analyses. A blood sample was collected from each specimen by puncturing the caudal vessel using needles and syringes containing 10% EDTA. Blood smears and panchromatic staining with May Grünwald-Giemsa-Wright (Ranzani-Paiva et al., 2013) were performed to observe the parasite structures. The samples were examined for hemoparasites under an optical microscope (Lumen LM3100) at 40x and 100x magnification and identified as described by Lom & Dyková (2006).

The fishes were anesthetized using tricaine methanesulfonate (MS-222), followed by euthanasia with neural myelotomy, necropsy and macroscopic observation of the kidney tissues, to check for the presence of cysts, lesions and parasites. During the necropsy, kidney fragments were visualized fresh between slides and coverslips, tissues with cysts were collected and analyzed by optical microscopy.

Parasite spores were recorded using a Moticam 2300 3.0 M camera with Motic images Plus 2.0 software attached to the microscope. The morphometric data of mature and fresh spores were obtained (μm) according to Matos et al. (2001) and analyzed as recommended by Lom & Arthur (1989).

## Results and Discussion

The *M. lippincottianus* specimens were 7.94 ± 0.54 cm in total length and 11.18 ± 1.54 g in body weight. The prevalence of *Myxobolus* spp. infecting the circulating blood of the fish collected in the Curiaú River was 36.36% (4/11) and 15 hemoparasites were found. *Myxobolus* spp. had a prevalence of 54.55% (6/11) in host's kidney tissue. However, clinical signs of infection, common to the genus, could not be observed in the parasitized fish.

**Figure 2:**
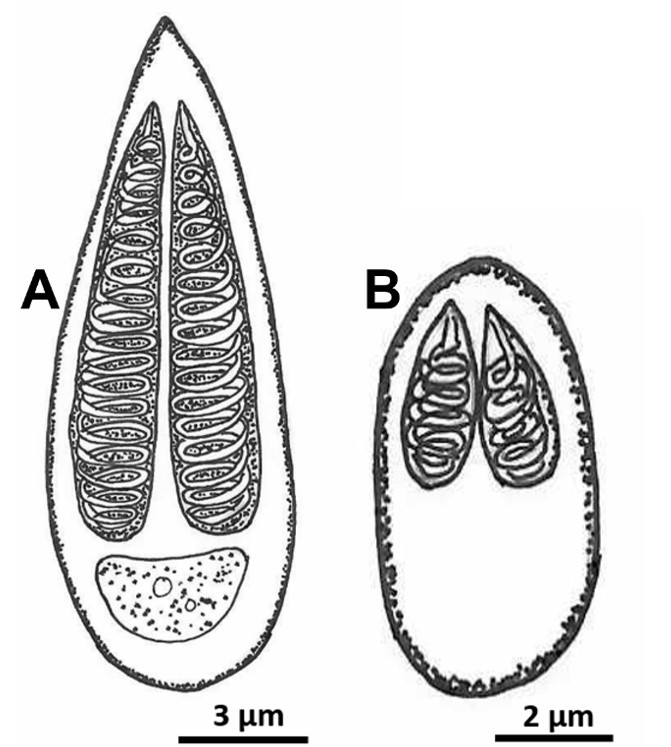
Illustrative drawings of the two *Myxobolus* spp. morphotypes: (A) drop-shaped; (B) oval-shaped. Hemoparasites in valve view.

*Metynnis lippincottianus* is a freshwater pelagic fish with a diversity of ectoparasites and endoparasites (Hoshino & Tavares-Dias, 2014), such as those of the subphylum Myxozoa. *Myxobolus* is a genus of the family Myxobolidae, belonging to the phylum Cnidaria and class Myxosporea, which has the largest number of members, with more than 900 species described in literature (Eiras et al., 2021). In this study, the myxospores had two pyriform polar capsules of equal size, bilateral symmetry, and binucleate sporoplasm, indicating that the identified parasites belonged to the genus *Myxobolus* (Figure 2).

**Figure 3:**
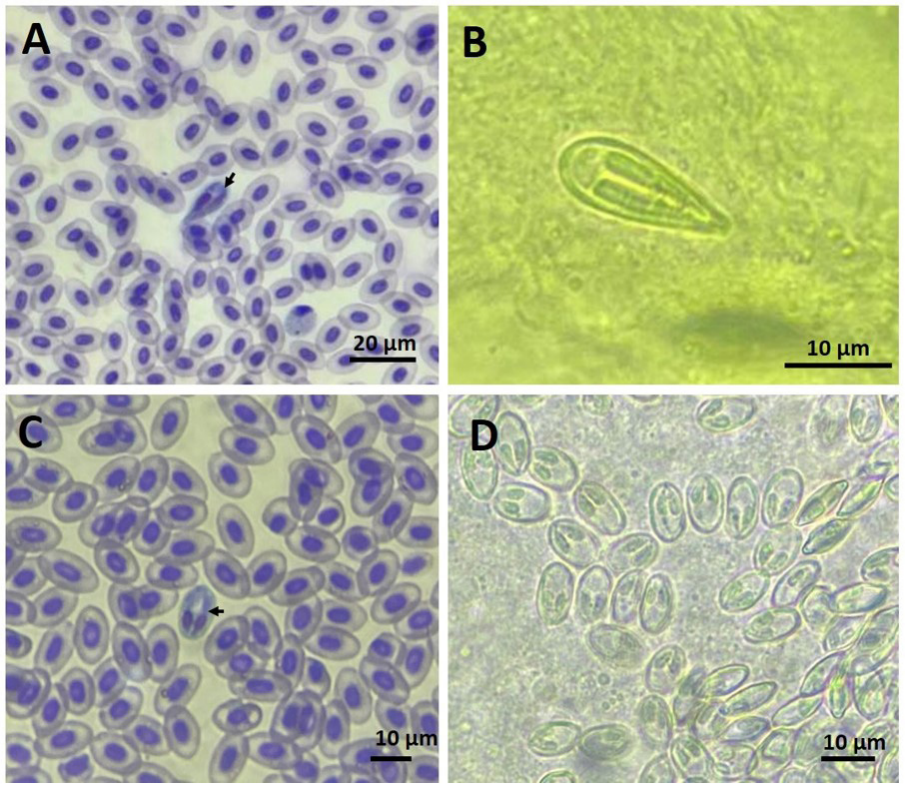
Photomicrograph of fresh *Myxobolus* spp. spores infecting *Metynnis lippincottianus* from the Curiaú River Environmental Protection Area, Amapá State, Eastern Amazon, Brazil. (A) and (B) Drop-shaped morphotype of *Myxobolus* spp. observed: (A) in the circulating blood (arrow); and (B) in the kidneys; (C) and (D) Oval-shaped morphotype of *Myxobolus* spp.: (C) in the blood (arrow); and (D) in the kidneys.

The morphometric survey revealed two morphotypes of *Myxobolus* spp., one elongated in the shape of a drop (M1) and the other oval (M2) (Figure 3). The M1 parasites measured 17.00 ± 1.49 µm in spore length and 6.00 ± 0.61 µm in width; the polar capsules present the same size with 15.00 ± 1.13 µm long and 2.40 ± 0.37 µm wide, with 16 to 18 turns of the polar filament (Figure 3A). The M2 spores were on average 11.01 ± 0.07 µm long and 4.08 ± 0.30 µm wide; the polar capsules present the same size and with 6.53 ± 0.41 µm long and 1.56 ± 0.21 µm wide, with 4 to 6 turns of the polar filament (Figure 3B).

The drop-shaped spores showed morphological characteristics similar to those of the *Myxobolus maculatus* found parasitizing the kidney of the Amazonian fish, *Metynnis maculatus* (Casal et al., 2002) (Table 1). In this study, we could not determine the species level of *Myxobolus* spp. because, in addition to methods based on morphology, morphometry, and biology (location in host tissue, morphology of sporogonic stages, and other developmental stages), use of precision devices, such as molecular tools was needed (Holzer et al., 2010; Fujimoto et al., 2013).

**Table 1:** Comparative measurements of *Myxobolus* spp. in *Metynnis lippincottianus* with other serrasalmids species of Brazil.

Parasite	Spore length	Spore width	Polar capsule length	Polar capsule width	Polar filament (number of coils)	References
*Myxobolus maculatus*	21.0 (9.7-23.0)	8.9 (7.9-9.5)	12.7 (11.8-13.8)	3.2 (3.0-3.6)	14-15	Casal et al. (2002)
*M. metynnis*	12.9-13.5	7.5-8.3	5.0–5.5	5.0-5.5	8-9	Casal et al. (2006)
*M. colossomatis*	11.8 (11.4-12.2)	6.9 (6.6-7.2)	6.0 (5.8-6.6)	6.0 (5.8-6.6)	7-8	Molnár & Békési (1993)
*M. colossomatis*	9.6 (9.1-10.28)	5.0 (4.6-5.1)	5.5 (4.9-6.3)	1.6 (1.3-1.7)	-	Maciel et al. (2011)
*Myxobolus* sp.	9.87 (8.9-9.8)	6.3 (5.4-6.7)	4.0 (4.3-5.31)	2.4 (2.0-2.9)	-	Úngari et al. (2021)
*Myxobolus* sp1.	17.0 (15.5-18.4)	6.0 (5.4-6.6)	15.0 (13.9-16.1)	2.4 (2.0-2.6)	16-18	Present study
*Myxobolus* sp2.	11.0 (11.1-11.4)	4.1 (3.8-4.4)	6.5 (6.1-6.9)	1.6 (1.4-1.8)	4-6	Present study

This group of parasites has an indirect and complex life cycle for two hosts, invertebrates, such as aquatic annelids, and intermediate vertebrates, mainly fish (Eszterbauer et al., 2015). Generally, the species of genus *Myxobolus* are morphologically characterized by their pyriform shape and presence of spores in their life cycle; they can be differentiated by the size of their polar capsules, number of polar filament turns, sporoplasm formation and valve formation (Lom & Arthur 1989; Fiala et al., 2015; De Araujo et al., 2018).

Further, organisms of the genus *Myxobolus* have two polar capsules, which originate from cells found inside the spore. The polar filaments have a helical shape and sporoplasm with a binucleate cell; the number of turns of the polar filament is important for characterizing and identifying the species. This cell contains several electrodense vesicles known as sporoplasmosomes (Casal et al., 2006).

In the Brazilian Amazon, the presence of myxosporidians has been recorded in *M. lippincottianus*, which the presence of *Henneguya* sp. has been reported and two morphotypes of *Myxobolus* sp. in the gills of this host (Carvalho et al., 2019). In work carried out by Carvalho et al. (2020), the presence of *Henneguya* sp. in 80% of the gills of *M. lippincottianus* caused hyperplasia and fusion of the gill lamellae. Both studies were carried out in the same collection area as this research.

In Brazil, only five records of *Myxobolus* spp. were reported in the fish blood (Table 2), and this work is the first record of the occurrence of *Myxobolus* spp. parasitizing the blood of *M. lippincottianus*. It is worth mentioning that this species of myxozoan has already been found in several fish organs, such as gills, blood vessels and caudal kidney (Cirkovic et al., 2010; Mabika et al., 2016; Manrique et al., 2017; Batueva, 2020; Maftuch et al., 2021; Silva et al., 2023), with the bloodstream being the least recurrent organ for *Myxobolus* sp. parasitism, which demonstrates the degree of relevance of this work.

**Table 2:** Occurrence of Myxozoa parasites in fish blood; *Myxobolus* sp1.: drop-shaped; *Myxobolus* sp2.: oval-shaped.

Host	Family	Parasite	Infection site	Collection site	References
*Carassius auratus*	Cyprinidae	*Myxobolus honghuensis*	Pharynx, pseudo branch, gill, brain, head kidney, kidney, liver, spleen, ovary, intestine and blood	Jiangsu, China	Yang et al. (2022)
*Tetragonopterus araguaiensis*; *Myloplus rubripinnis*; *Pygocentrus nattereri*	Characidae; Serrasalmidae; Serrasalmidae;	*Myxobolus* spp.	Blood	Goiás and Mato Grosso, Brazil	Úngari et al. (2021)
*Colossoma macropomum*	Serrasalmidae	*Myxobolus colossomatis*	Blood	Amazonas, Brazil	Maciel et al. (2011)
*Salmo trutta*	Salmonidae	*Chloromyxum schurovi; Chloromyxum truttae; Tetracapsuloides bryosalmonae and Sphaerospora truttae*	Gills, renal tubules, gall bladder, brain, heart and blood	Scotland, United Kingdom	Holzer et al. (2006)
*Baryancistrus* sp.	Loricariidae	*Sphaerospora* sp.	Renal tubules and blood	Amazonas, Brazil	Paperna & Di Cave (2001)
*Alburnus alburnus*; *Leuciscus aspius*; *Blicca bjoerkna*; *Abramis ballerus*; *Rutilus rutilus*; *Scardinius erythrophthalmus*	Cyprinidae	*Sphaerospora* spp.	Kidney and blood	Hungary	Baska & Molnar (1988)
*Rutilus rutilus*; *Gobio gobio*; *Tinca tinca*	Cyprinidae	*Sphaerospora* sp.	Kidney and blood	Prague, Czechoslovakia	Lom et al. (1985)
*Metynnis lippincottianus*	Serrasalmidae	*Myxobolus* sp1. and *Myxobolus* sp2.	Kidney and blood	Amapá, Brazil	Present study

According to Holzer et al. (2006), the presence of myxosporeans may be related to the use of blood as a means of transportation and a channel for parasite proliferation in the target organs, the kidney and the gall bladder, thus demonstrating a possible justification for the evidence obtained in this study. Maciel et al. (2011), state that blood samples from fish must be evaluated to report the presence of myxozoans, since in their study the possibility of blood contamination due to the presence of this parasite, whether in the form of plasmodium, free spores in the mucosa, epithelium or connective tissues that were accessed during the puncture of the caudal vein.

In this study, spores of *Myxobolus* spp. were identified in the fresh caudal kidney, and the morphometric data of the spores found in the blood converge with those observed in the kidney. The spores of *Myxobolus* spp. develop in the internal organs from where mature myxospores are transported via the bloodstream to reach the target organ, caudal kidney (Molnár et al., 2009; Bjork & Bartholomew, 2010 and Manrique et al., 2017). The observed results reinforce the hypothesis that *Myxobolus* spp. complete their sporogonic development or with release into the environment from the host urine. Sipos et al. (2018) proved through experimental results that *Myxobolus cerebralis* is found in blood during the initial stage of host infection, and that the intensity of infection in the blood decreases over time and with the growth phase.

Detailed studies on the morphology of *Myxobolus* spp. are important for understanding the biology and epidemiology of these parasites, as well as for developing effective diagnostic and treatment methods in cases of parasite outbreaks. Further, the morphology of spores, polar capsules and the number of valves can provide valuable information for identifying and characterizing different *Myxobolus* species that affect fish in the Amazon region.

The results obtained and compared with the literature demonstrated in this study allowed us to conclude that *Myxobolus* spp. are parasites of *M. lippincottianus* (Osteichthyes: Serrasalmidae) originating from the Curiaú River and although the fishes analyzed were apparently healthy, studies like this are sound, they are essential to protect the health and survival of fish populations and to maintain the health of aquatic ecosystems as a whole. So, considering the relevance and importance of this study, further research is needed to obtain more information on the life cycle in this intermediate host and the epidemiological potential linked to the infection of *Myxobolus* spp. in the circulating blood and kidney of *M. lippincottianus*.
